# Rigid soles improve balance in beam walking, but improvements do not persist with bare feet

**DOI:** 10.1038/s41598-020-64035-y

**Published:** 2020-05-06

**Authors:** Meghan E. Huber, Enrico Chiovetto, Martin Giese, Dagmar Sternad

**Affiliations:** 1 Department of Mechanical Engineering, Massachusetts Institute of Technology, Cambridge Massachusetts, USA; 2 Section for Computational Sensomotorics, Department of Cognitive Neurology, Hertie Institute for Clinical Brain Research, Centre for Integrative Neuroscience, University Clinic Tübingen, Tübingen, Germany; 3 Departments of Biology, Electrical and Computer Engineering, and Physics, Northeastern University, Boston Massachusetts, USA

**Keywords:** Learning and memory, Motor control

## Abstract

Maintaining balance while walking on a narrow beam is a challenging motor task. One important factor is that the foot’s ability to exert torque on the support surface is limited by the beam width. Still, the feet serve as a critical interface between the body and the external environment, and it is unclear how the mechanical properties of the feet affect balance. This study examined how constraining the motion of the foot joints with rigid soles influenced balance performance when walking on a beam. We recorded whole-body kinematics of subjects with varying skill levels as they walked on a narrow beam with and without wearing flat, rigid soles on their feet. We computed changes in whole-body motion and angular momentum across the two conditions. Results showed that walking with rigid soles improved balance performance in both expert and novice subjects, but that improvements in balance performance with rigid soles did not affect or transfer to subsequent task performance with bare feet. The absence of any aftereffects suggested that the improved balance performance resulting from constraining the foot joints by a rigid sole was the result of a mechanical effect rather than a change in neural control. Although wearing rigid soles can be used to assist balance, there appears to be limited benefit for training or rehabilitation of balance ability.

## Introduction

Whether walking over rocks or across logs, humans have remarkable ability to maintain balance while navigating irregular and uneven terrain^1,2^. In fact, healthy humans are so proficient in their ability to balance that some turn to walking on a thin wire or a slack line to truly challenge their skills. While there has been prolific research on the control of postural balance over the past decades, this work has largely focused on understanding how humans maintain balance during quiet standing on solid ground^3–8^. Despite many insights into the limits of postural balance, it is still an open question how the central nervous system controls the highly redundant and extremely complex architecture of the body to maintain balance during more realistic locomotion, especially in challenging environments.

A paradox of human motor control is that while the human body is immensely complex (e.g., large number of degrees of freedom, intersegmental dynamics, long time delays, ubiquitous sensorimotor noise, nonlinear muscle properties), the overt behavior is often surprisingly simple in structure. Thus, low-dimensional models, derived by compressing the number of degrees of freedom in the body, have been remarkably competent at describing human balance. For example, an inverted pendulum can adequately capture much of the behavior that humans exhibit during quiet stance^8^. When the base of support is reduced, such as in the case of standing on a narrow beam, adding a second linkage to make a double-inverted pendulum has proven sufficient^8–10^. In a recent study, Chiovetto *et al.*^11^ revealed that the segmental dynamics of the body, quantified by angular momentum of each segment about the top of the beam, exhibited a lower-dimensional structure compared to the structure in relative joint angle kinematics. Using principal component analysis, a single component could explain about 90% of the variance in the angular momentum of all body segments and was highly similar across individuals. Unlike several prior studies on standing and walking on a beam, Chiovetto *et al.*^11^ allowed participants to freely move their arms during the experiment with the goal to look at the full complexity of the realistic behavior. How does the nervous system control the high-dimensional architecture of the entire body to generate such low-dimensional patterns?

A critical aspect of maintaining balance is managing the physical interaction between the body and its external environment. Because the feet serve as interfaces through which the body and ground simultaneously act upon each other, they play a pivotal role in maintaining balance. As seen in the development of prosthetics, the mechanical properties of the foot can significantly influence balance behavior^12–14^. And yet, how the complex architecture of the human feet contributes to balance performance is still poorly understood. Each foot consists of many articulated, rigid segments which are surrounded by compliant, heterogeneous tissue, making it difficult to accurately measure and model the subtle coordinated behavior of the foot^15–17^. Paradoxically, most models of human balance performance drastically simplify the foot. In the inverted pendulum models of standing balance, the foot is typically reduced to a rigid segment attached to the ground acted upon by an ideal torque source at the ankle. Thus, the foot’s influence on human balance performance, particularly during walking, remains understudied.

**Figure 1:**
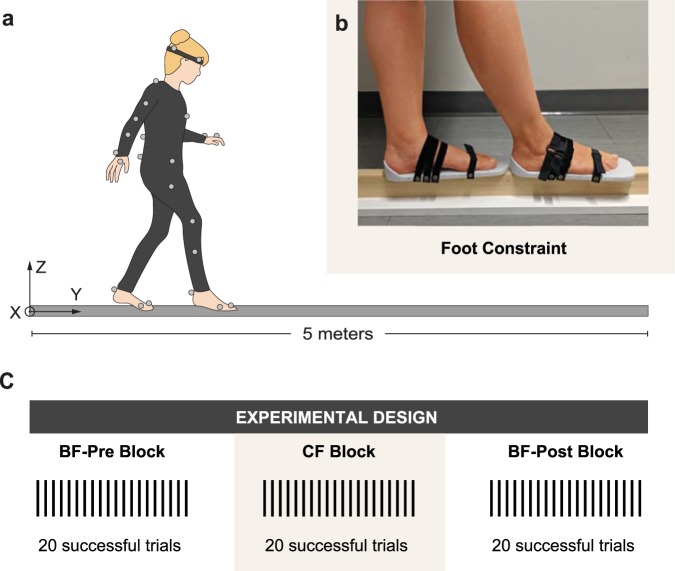
(**a**) Subjects walked on a narrow beam (3.4 cm wide by 5 m long) wearing reflective markers for 3D kinematic data acquisition. One traversal of the entire beam without stepping off was considered a successful trial. (**b**) Flat, rigid soles were attached to the bottom of the subjects’ feet to constrain the motion of the foot joints and toe bending. The soles were attached with hook-and-loop straps and reinforced with tape. Ankle motion was not constrained. (**c**) Subjects completed 20 successful trials in each of three blocks: Bare Feet (BF)-Pre, Constrained Feet (CF), and Bare Feet (BF)-Post.

The aim of this study was to understand how constraining the motion of the foot joints with rigid soles affects one’s ability to maintain mediolateral (ML) balance when walking on a narrow beam (Fig. 1a). Like Chiovetto *et al*., this study again allowed subjects to freely move their arms as in real life. Motion of the joints in both feet (e.g., tarsometatarsal, metatarsophalangeal, and interphalangeal joints) were constrained by attaching a flat, rigid sole to the bottom of each foot (Fig. 1b). The rigid soles also prevented any bending at the midfoot and torsion on the long axis of the foot. Importantly, plantarflexion/dorsiflexion and inversion/eversion ankle motion were not constrained.

On the one hand, the highly flexible foot may be critical for actively sensing and controlling the physical interaction between the foot and the beam. From observing a person standing on a beam, it is evident that the numerous joints in the foot including the soft tissue move and conform to the beam presenting a rich source of haptic and proprioceptive information. In addition, Sawers *et al.*^18^ found that dancers used more muscle synergies (i.e., an increased set of available whole-body actions) to maintain balance when walking barefooted on a beam compared to novices. This strongly suggests that limiting kinematic degrees of freedom may reduce the movements available to withstand perturbations and maintain upright balance. Similarly, constraining joint motion in the feet could impair balance performance and worsen performance in the beam walking task (***Hypothesis 1a***). An alternative argument, however, is equally plausible. For example, Robbins *et al.*^19^ found that elderly men improved beam walking when they wore shoes with hard, thin soles. They stepped off the beam less frequently compared to performing the task with bare feet or shoes with softer soles. Constraining the foot joints would make the feet act as rigid, flat segments. This could reduce the dynamics at the foot-beam interaction port, which could increase contact stability^20^ (i.e., the tendency to return to an equilibrium posture with contact between the feet and the flat surface of the beam) and thereby improve balance performance. For instance, a flat, rigid sole atop a flat surface is statically stable. Hence, an alternative expectation is that rigid soles positively affect balancing performance (***Hypothesis 1b***).

Changing the mechanics of the feet with rigid soles could also cause subjects to adapt their neural control strategy with practice. If so, this altered strategy might still influence balance performance after the rigid soles are removed. For instance, if the rigid soles led to worse performance, we would expect subjects to adapt their control strategy to maintain balance as best as possible. When the rigid soles were removed, they would relatively quickly return to their original control strategy as the adapted strategy would no longer have any benefit (***Hypothesis 2a***). This scenario corresponds to many studies on the adaptation on the control of reaching where, for example, adaptation to a perturbing force field only persists as short-term aftereffects as the adapted strategy is no longer functional when the perturbation is removed^21–23^. This aftereffect is generally seen as evidence that the internal model of the environment and, consequently, some neural control mechanisms have changed. In contrast, wearing rigid soles may also lead to improved balance performance, again reflecting a change in neural control strategy that exploits the altered mechanics. For instance, the soles could provide physical guidance^24,25^ allowing subjects to explore and adopt new strategies that they might not feel comfortable or safe when performing the task with bare feet. After removing the soles, we would expect that this acquired strategy would persist as it is a functional adaptation that endows benefits also in the original environment (***Hypothesis 2b***). Such a scenario would indicate that the soles could be used as a teaching aid to accelerate learning to balance. In addition, there is a third feasible scenario: humans do not alter their control policy when the rigid soles are attached to their feet. For example, any change in performance may only be due to the change in the foot mechanics without any change in their control policy. In this case, we would expect practice with constrained feet to have no influence on subsequent performance with bare feet (***Hypothesis 2c***). While the two previous scenarios have been discussed in the literature, this third option is an under-recognized possibility. By assessing how practice of the beam-walking task with rigid soles influences subsequent balance behavior with bare feet, we gain insight not only into the role of the complex architecture of the foot on the neural control of balance, but also whether rigid soles may be a suitable intervention for either assisting or rehabilitating impaired balance behavior.

This study investigated how constraining the feet affected mediolateral (ML) balance performance in beam walking for young individuals with varying levels of prior balance training. We evaluated the two hypotheses using metrics derived from measured whole-body kinematics. Note that measured balance performance and the ability to balance needs to be distinguished. Here, we refer to balance ability or proficiency when we mean one’s ability to maintain an upright posture. To assess individuals that could be assumed to have basic and advanced balance abilities, we included both novices and skilled gymnasts. In contrast, we refer to balance performance, or sometimes only ‘balance’, as to what is measured and quantified as ‘effective balance’. We calculated mediolateral (ML) balance during beam walking compared to performing the task with bare feet. Previous work has shown that the lower variability of the velocity of the center of mass (COM-V) in the ML-direction is a good indicator of skilled balance performance^11^. Hence, impaired balance performance is indicated by an increased variability of the velocity of the center of mass (COM-V) in the ML-direction. In addition, we expect impaired balance performance to be evident as increased whole-body angular momentum (WB-AM) about the beam axis; improved balance performance would show the opposite trend. To evaluate whether practice with constrained feet affected performance after removing the rigid soles, we tested subjects walking with bare feet not only before, but also after walking with rigid soles. In addition to testing the hypotheses, further analyses of whole-body coordination were conducted to shed light on how constraining the foot by wearing rigid soles influenced ML-balance during beam walking.

In overview, the results showed that constraining the feet improved ML-balance in the beam walking task (***Hypothesis 1b****)*. However, subsequent task performance with bare feet did not benefit from this improved balance with rigid soles (***Hypothesis 2c***). Additional analyses showed that the reduction of WB-AM when wearing the rigid soles resulted from a decrease in the angular momentum of most individual limb segments. Moreover, the contribution of ankle torque relative to hip torque was increased when the feet were constrained. Together, we conclude that wearing rigid soles improved balance performance due to an increase in contact stability between the foot and the beam. This suggests a mechanical effect rather than a change in neural strategy.

## Results

Seven healthy subjects took part in the experiment. Their prior balance training ranged from none to several years in competitive gymnastics. In each trial, subjects were instructed to walk the length of a narrow beam (3.4 cm wide and 5 m long) without stepping off the beam (Fig. 1a). They were allowed to freely move their arms. A trial was deemed successful if the subject did not step off before reaching the end of the beam; otherwise the trial was declared a failed trial. Subjects had to complete 20 successful trials in each of the following three blocks: The first block consisted of 20 successful trials with bare feet (BF-Pre block), followed by 20 successful trials with constrained feet (CF block, Fig. 1b), and another 20 successful trials with bare feet (BF-Post block) (Fig. 1c).

### Number of failed trials

**Figure 2:**
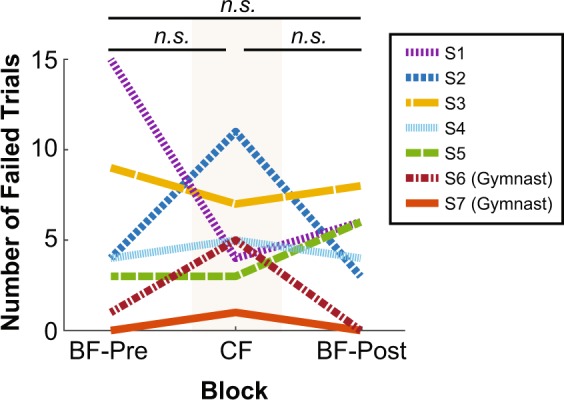
Number of failed trials of individual subjects in each block. “*n.s*.” indicates no significant pairwise difference. Subjects 6 and 7 were trained gymnasts, and the remaining subjects were novices. The colors and line styles for each subject are consistent across results figures.

To gauge if constraining subjects’ feet affected their ability to accomplish the beam-walking task, we first compared the number of failed trials in each of the three blocks. A one-way within-subjects analysis of variance (ANOVA) revealed that foot condition (BF-Pre, CF, BF-Post) did not have a significant effect on the number of failed trials (F_2,12_ = 0.38, p = 0.69) (Fig. 2). On average, subjects failed in approximately 4–5 trials in each block. As expected, performance across subjects varied, determined in part by their prior balance training. Subjects who exhibited the best performance (shown in red and orange in Fig. 2) were trained gymnasts. As the results below show, the cohort presented a sufficient spectrum of balance abilities that allowed more general conclusions.

### Example data

**Figure 3:**
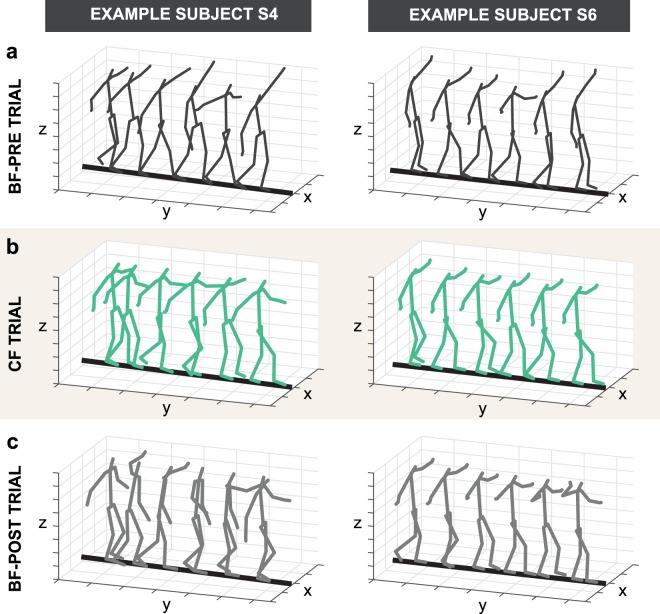
Representative stick figures of two example subjects walking across the beam constructed from 3D kinematic data during one representative trial from each block: (**a**) Barefoot-Pre (BF-Pre), (**b**) Constrained foot (CF), (**c**) Barefoot-Post (BF-Post). The example subject on the left is a novice shown by the blue line (S4) and the example subject on the right is the trained gymnast shown by the red line (S6) in Fig. 2.

**Figure 4:**
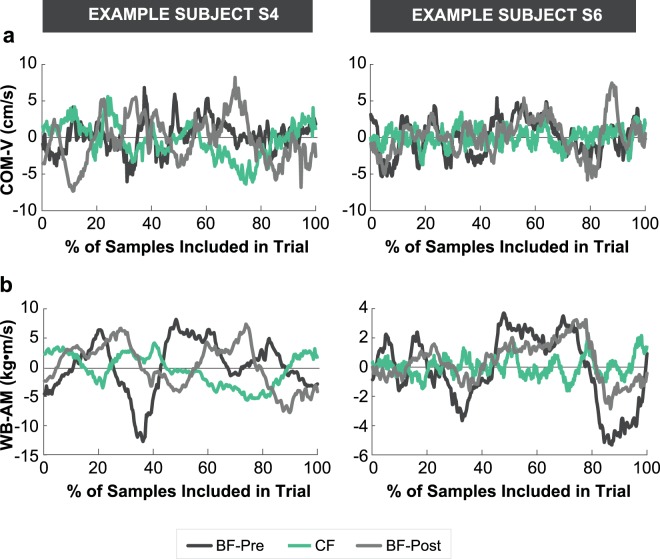
Time series of (**a**) velocity of the center of mass (COM-V) and (**b**) whole-body angular momentum (WB-AM) of representative trials in each block from the two example subjects. Note that the scale of the figures showing WB-AM are different as the magnitude of the metric is considerably smaller in the expert subject. The trials shown here are the same as in Fig. 3. Grey, green and black lines refer to trials in the barefoot pre (BF-Pre) block, the constrained foot (CF) block and the barefoot post (BF-Post) block, respectively. Only the middle 67% of samples of the entire trial (indicated in this figure as 100% of included samples) were used for calculating the root-mean-square (RMS) of each dependent measure in each trial.

**Figure 5:**
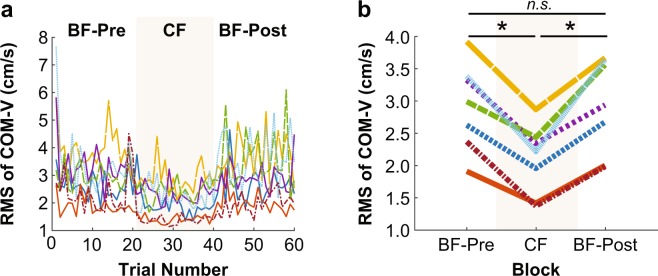
Root mean square (RMS) of the velocity of the center of mass (COM-V) of individual subjects (**a**) across all trials and (**b**) averaged within each block. An asterisk represents significance at p < 0.05, and “*n.s*.” indicates no significant difference.

**Figure 6:**
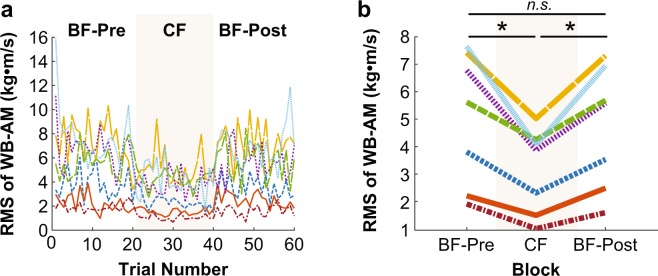
Root mean square (RMS) of whole-body angular momentum (WB-AM) of individual subjects (**a**) across all trials and (**b**) averaged within each block. An asterisk represents significance at p < 0.05, and “*n.s*.” indicates no significant difference.

Even though constraining the subjects’ feet did not require more attempts to accomplish the overall task goal, analysis of more fine-grained measures revealed that it did significantly influence their balance performance. Figure 3a–c displays the series of whole-body postures of two representative subjects during a typical trial in each of the three blocks. For reference, data from example subject S4 is shown in light blue in other result figures; example subject S6, who was trained in gymnastics, is shown in dark red. Subjects displayed not only large trunk movements, but also large and variable movements of both arms. Importantly, these body movements were visibly reduced in the CF block. Figure 4a–b shows the time series of the center of mass velocity (COM-V) and whole-body angular momentum (WB-AM) for the corresponding trials shown in Fig. 3a–c. Performance in each trial was summarized by taking the root-mean-square (RMS) of these dependent measures in the middle 67% (i.e., two-thirds) of the trial. For both subjects, the amplitude of the COM-V and WB-AM signals were decreased in the CF block (Figs. 4–6**)**, reflecting the decrease in body movement observed in Fig. 3b.

### Center of mass velocity (COM-V)

As demonstrated in prior work^11^, the RMS of COM-V in the ML direction of each trial was sensitive to differences in balance training. Note this is not necessarily always the case. For instance, there are scenarios, such as in competitive gymnastics, during which a talented balancer may intentionally generate larger whole-body movements as a demonstration of skilled balance ability. In the present study, however, the experienced subjects, shown in red and orange in Fig. 5a, exhibited lower velocity of the center of mass (COM-V). Consistent with the qualitative observations from Figs. 3 and 4a, a one-way within-subjects ANOVA on RMS of COM-V revealed a significant effect of block (F_2,12_ = 32.99, p = 0.000031) (Fig. 5a,b). Planned comparisons showed a significant reduction in RMS of COM-V from the BF-Pre block (M = 2.93 cm/s, SD = 0.68 cm/s) to the CF block (M = 2.09 cm/s, SD = 0.55 cm/s) (t_6_ = 8.40; p = 0.00016). This was followed by a significant increase from the CF block to the BF-Post block (M = 2.92 cm/s, SD = 0.74 cm/s) (t_6_ = −6.93; p = 0.00045). These results indicated that constraining subjects’ feet significantly improved their ML balance performance (***Hypothesis 1b***). Moreover, there was no difference in the mean RMS of COM-V between the BF-Pre block and the BF-Post block (t_6_ = 0.06, p = 0.95) (Fig. 5b). This was also a first indication that there was no benefit transferred from wearing rigid soles to walking barefoot thereafter (***Hypothesis 2c***). Even though subjects varied in their ability to perform the task, the decrease in the RMS of COM-V during the CF block was seen across all subjects.

To further scrutinize ***Hypothesis 2a-c***, the last successful trial of the BF-Pre block was compared with the first trial of the BF-Post block. There was no significant difference between the last trial of the BF-Pre block (M = 2.80 cm/s, SD = 0.66 cm/s) and the first successful trial of the BF-Post block (M = 2.62 cm/s, SD = 0.71 cm/s) (t_6_ = 0.52, p = 0.62). This indicated an immediate return to baseline performance after the rigid soles were removed (Fig. 5a). Thus, practice with constrained feet did not influence subjects’ balance performance with bare feet (***Hypothesis 2c***).

### Whole-body angular momentum (WB-AM)

We also examined how performing the balance beam task with rigid soles influenced subjects’ whole-body angular momentum (WB-AM). When walking on the beam, the body is subject to ground reaction forces acting on the feet. These external forces induce considerable changes in the body’s WB-AM. Specifically, the measure of WB-AM quantified the angular momentum of a subject’s body with respect to the beam. We quantified WB-AM with respect to the beam axis, rather than the body’s center of mass or head position for two reasons: First, the beam was fixed and thus provided an inertial reference frame. Second, our prior work revealed that the structure of AM was less complex when quantified about the beam axis^11^.

Figure 6a show the time series of RMS of WB-AM across blocks and trials, indicating a similar decrease in WB-AM during the CF block as for COM-V. The same one-way ANOVA similarly rendered a significant effect of block on the RMS of WB-AM (F_2,12_ = 21.73, p < 0.001) (Fig. 6b). Planned comparisons revealed that constraining the foot had a similar effect on RMS of WB-AM as it did on COM-V. The RMS of WB-AM significantly decreased from the BF-Pre block (M = 5.07 kg·m^2^/s, SD = 2.40 kg·m^2^/s) to the CF block (M = 3.17 kg·m^2^/s, SD = 1.52 kg·m^2^/s) (t_6_ = 4.69, p = 0.0034), and then significantly increased from the CF block to the BF-Post block (M = 4.75 kg·m^2^/s, SD = 2.21 kg·m^2^/s) (t_6_ = −5.33, p = 0.0018). There was no difference in RMS of WB-AM between the BF-Pre block and the BF-Post block (t_6_ = 1.71, p = 0.14). As above, there was also no difference between the last successful trial of the BF-Pre block (M = 4.01 kg·m^2^/s, SD = 2.08 kg·m^2^/s) and the first successful trial of the BF-Post block (M = 4.67 kg·m^2^/s, SD = 2.158 kg·m^2^/s) (t_6_ = −0.86, p = 0.42) (Fig. 6a). Again, these results indicate that constraining subjects’ feet significantly improved their ML balance performance (***Hypothesis 1b***), but the improved performance with constrained feet did not transfer or influence subjects’ subsequent performance with bare feet (***Hypothesis 2c***).

To further understand how WB-AM was reduced, we also examined how angular momentum (AM) of the individual body segments changed when wearing rigid soles. As WB-AM was calculated as the sum of AM from individual body segments about the beam, it could have been lowered in several different ways. For example, WB-AM could have been decreased by reducing the AM of either some or all body segments by changing the direction of segments’ AM relative to each other, or even a combination of both. Thus, we next assessed how wearing rigid soles influenced the spatiotemporal patterns of the individual body segments in their contribution to WB-AM.

### Angular momentum (AM) of individual body segments

**Figure 7:**
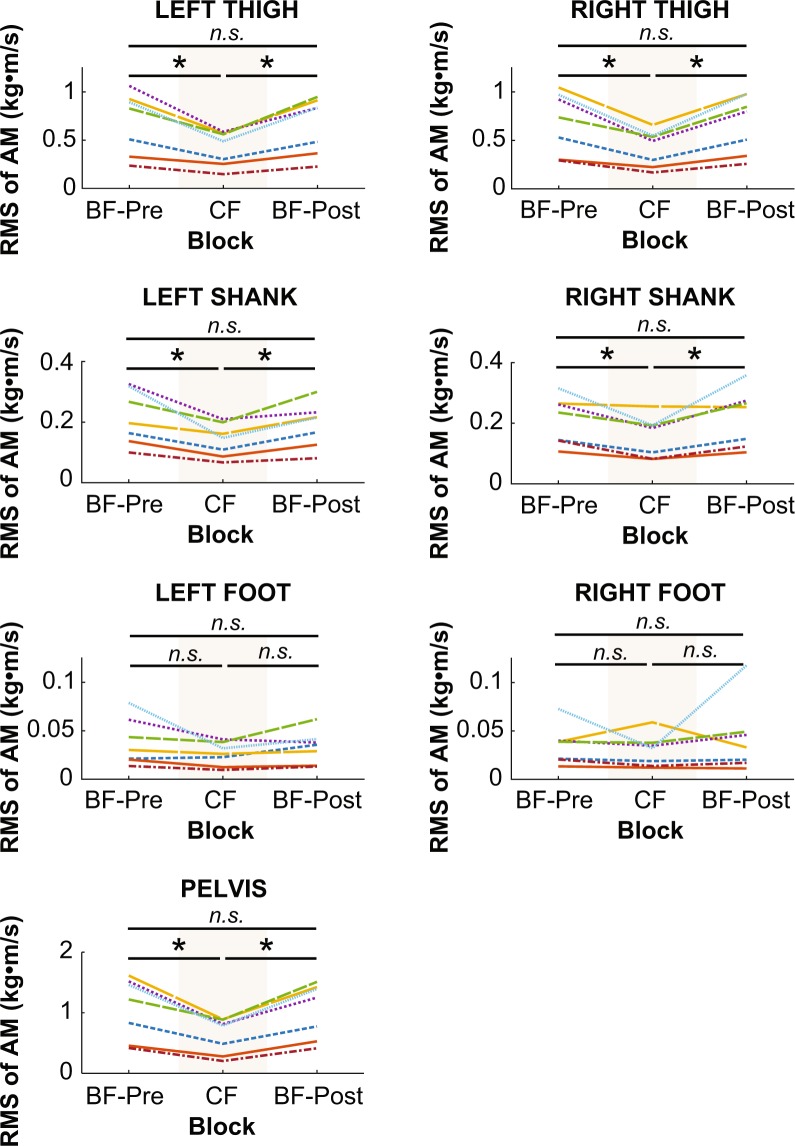
Root mean square (RMS) of lower-body segment angular momentum (AM) of individual subjects averaged within each block. An asterisk represents significance at p < 0.05, and “*n.s*.” indicates no significant difference.

**Figure 8:**
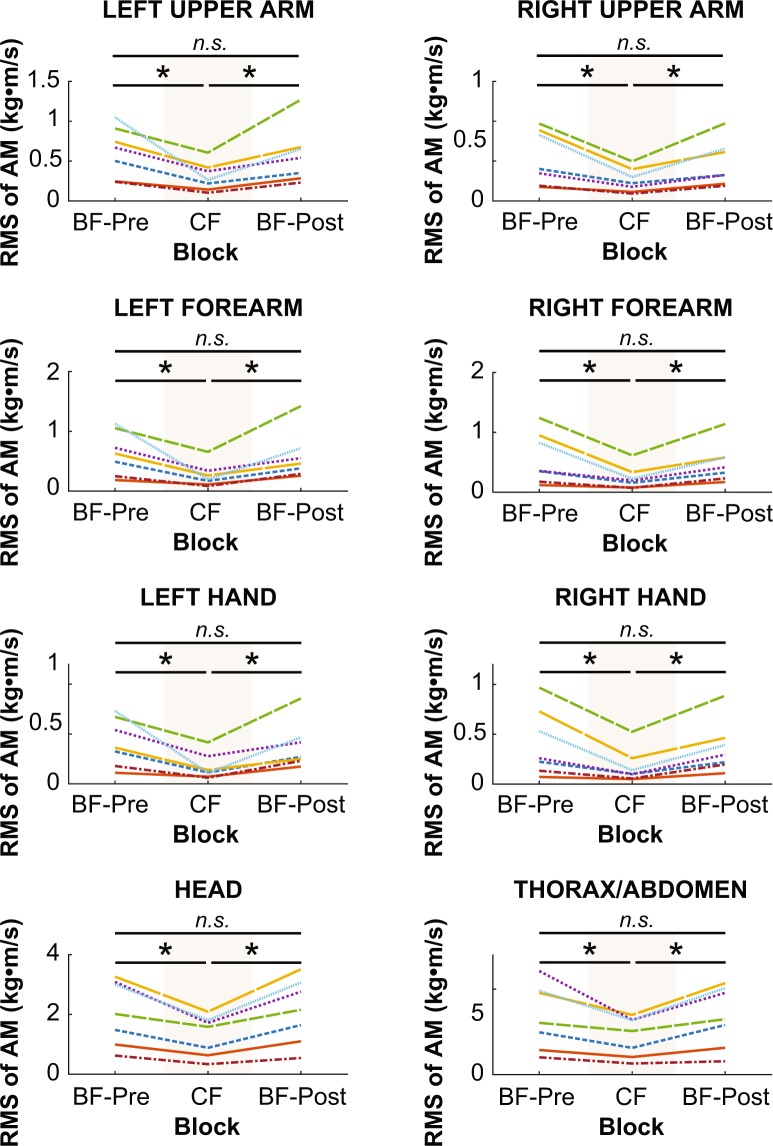
Root mean square (RMS) of upper-body segment angular momentum (AM) of individual subjects averaged within each block. An asterisk represents significance at p < 0.05, and “*n.s*.” indicates no significant difference.

For each of the 15 body segments, a one-way within-subjects ANOVA was conducted on the RMS of each segment’s AM. The results of each ANOVA are detailed in Table 1 and illustrated in Figs. 7–8. To summarize, the effect of block on each segment’s AM was significant, except for the left and right feet. When significant, planned comparisons revealed that the effect of block on each segment’s AM was similar to its effect on WB-AM. Each segment’s AM significantly decreased from the BF-Pre block to the CF block (ps > 0.014) and then subsequently increased from the CF block to the BF-Post block (ps < 0.024). There were no significant differences between AM in the BF-Pre and BF-Post blocks (ps > 0.14). Hence, the reduction in WB-AM when wearing rigid soles was due in large part to a reduction in each segment’s contribution to WB-AM. It was not the result of reduced AM from a single large segment, for example.

**Table 1:** One-way within-subjects ANOVA results on individual body segment AM with block as independent variable.

Segment #	Dependent Measure	dF	SS	F	p
1	Pelvis AM	2	0.89	22.39	0.000089*
2	Right Thigh AM	2	0.32	22.85	0.000081*
3	Left Thigh AM	2	0.31	18.30	0.00023*
4	Right Shank AM	2	0.16	9.69	0.0031*
5	Left Shank AM	2	0.021	9.93	0.0029*
6	Right Foot AM	2	0.00053	0.87	0.45
7	Left Foot AM	2	0.00054	2.16	0.16
8	Head AM	2	2.96	20.22	0.00014*
9	Thorax/Ab AM	2	6.13	10.92	0.0020*
10	Right Upper Arm AM	2	0.31	12.35	0.0012*
11	Left Upper Arm AM	2	0.41	8.71	0.0046*
12	Right Forearm AM	2	0.43	10.60	0.0022*
13	Left Forearm AM	2	0.58	9.29	0.0037*
14	Right Hand AM	2	0.23	11.49	0.0016*
15	Left Hand AM	2	0.25	10.16	0.0026*

*statistically significant

### Correlation of upper- and lower-body angular momentum (CORR-AM)

**Figure 9:**
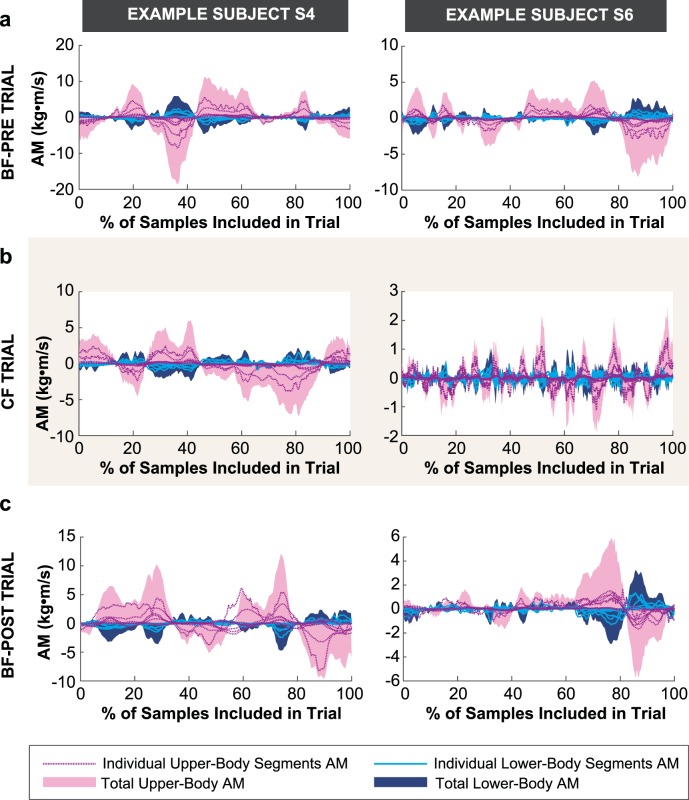
Time series of individual body segment angular momentum (AM), total upper-body angular momentum (UB-AM), and total lower-body momentum (LB-AM) of representative trials from two example subjects in each block: (**a**) Barefoot-Pre (BF-Pre), (**b**) Constrained foot (CF), (**c**) Barefoot-Post (BF-Post). Only the middle 67% of samples (indicated as 100% of included samples in this figure) were used for calculating the root-mean-square (RMS) of individual segment AM measure and correlation between UB-AM and LB-AM (CORR-AM) in each trial.

**Figure 10:**
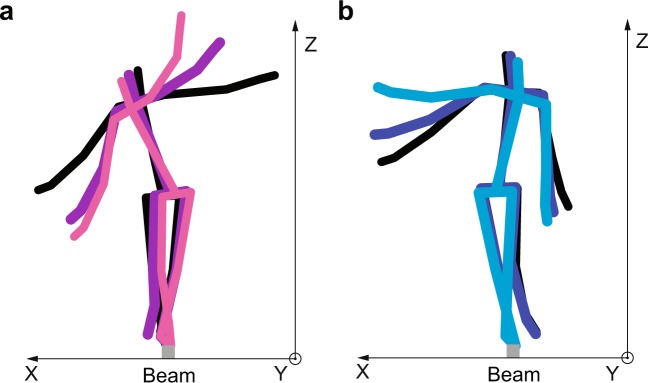
Representative stick figures of example subject S4 walking along the beam shown in the frontal plane. The color of the skeletons becomes lighter as the subject progresses forward along the beam axis (i.e., y-axis). (**a**) When the lower-body segments rotated clockwise about the beam, the upper-body segments rotated counterclockwise about the hip. (**b**) When the lower-body segments rotated counterclockwise about the beam, the upper segments rotated clockwise about the hip.

Figure 9 displays the time series of the individual body segment angular momenta (AM), the total upper-body angular momentum (UB-AM), and the total lower-body momentum (LB-AM) of representative trials from the same two exemplary subjects as above. As can be seen in all trials, the upper-body segments (head, thorax, upper arms, lower arms, and hands) generated AM in opposite direction to the AM generated by the lower-body segments (pelvis, thighs, shanks, and feet). To examine if the coordination of upper-body and lower-body AM contributions were affected by constraining the foot, we computed the correlation between the sum of AM of lower-body segments (LB-AM) and the sum of AM of upper-body segments (UB-AM) for each trial; we refer to this metric as CORR-AM. Note that three outlier trials (0.7% of all trials) were omitted from this analysis as the CORR-AM values were uncharacteristically low. Consistent with the representative data shown in Fig. 9, upper-body AM and lower-body AM were highly anticorrelated as the overall mean of CORR-AM across all conditions was −0.88 (SD = 0.05). As illustrated in Fig. 10, rotation of the upper-body segments about the hip was opposite to that of the lower-body segments about the beam. This suggests that subjects used a “hip-dominant” strategy to maintain balance^26^.

**Figure 11:**
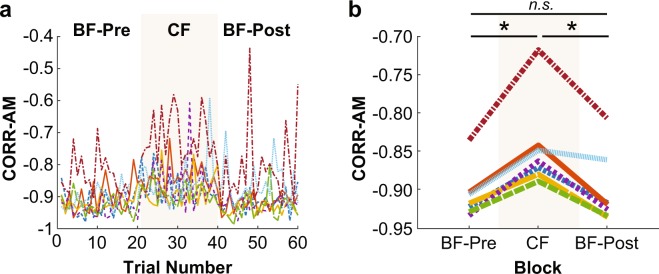
Correlation of the angular momentum of upper body and lower body (CORR-AM) of individual subjects (**a**) across all trials and (**b**) averaged within each block. An asterisk represents significance at p < 0.05, and “*n.s*.” indicates no significant difference.

A one-way within-subjects ANOVA on CORR-AM found a significant effect of block (F_2,12_ = 22.75, p = 0.000083) (Fig. 11a-b). The CORR-AM became less anticorrelated from the BF-Pre block (M = −0.89, SD = 0.03) to the CF block (M = −0.84, SD = 0.06) (t_6_ = −4.89, p = 0.0027) and then returned to more anticorrelation from the CF block to the BF-Post block (M = −0.90, SD = 0.04) (t_6_ = 5.45, p = 0.0016). There was no difference in CORR-AM between the BF-Pre block and the BF-Post block (t_6_ = 0.85, p = 0.43) (Fig. 11b). When focusing on the transition between the foot conditions, there was also no significant difference between the last successful trial of the BF-Pre block (M = −0.88, SD = 0.06) and the first successful trial of the BF-Post block (M = −0.94, SD = 0.03; t_6_ = 1.84, p = 0.12) (Fig. 11a). Interestingly, the upper-body AM and lower-body AM were less correlated when the foot was constrained, even though balance performance was improved. Consistently, the trained gymnasts (red and orange traces in Fig. 11a,b) also had the least anticorrelation between upper and lower body AM.

## Discussion

The two-fold goal of this study was to determine how constraining joint motion of the feet influenced whole-body coordination and neural control to maintain balance in a challenging beam walking task. In support of **Hypothesis 1b**, our results showed that wearing flat rigid soles improved balance immediately, i.e., in the first trial, as indicated by a reduction in the center of mass movement and whole-body angular momentum (RMS of COM-V and WB-AM). This finding is in accordance with Robbins *et al.*^19^ who showed balance performance in older individuals improved when wearing rigid-soled shoes. However, that study only measured balance performance by the number of an individual stepped off the beam. As our study showed, this is only a course-grained metric that does not sufficiently capture all effects of rigid soles. Further assessment of the transfer of improved balance from the rigid soles to barefoot walking revealed that rigid soles may not be effective for training or rehabilitating balance (**Hypothesis 2c**). Improved balance performance did not persist when walking with bare feet. This lack of transfer from constrained feet to barefoot walking suggests that the effect of rigid soles was mechanical and that subjects did not alter their control strategy in response to the foot motion constraint.

These results suggest that practice with rigid soles is unlikely to serve as an intervention strategy to rehabilitate balance control. A therapeutic intervention should not only temporarily enhance the performance, but also lead to long-lasting improvements in task performance under normal conditions^27^. Such longer-term persistence implies that the intervention also affected neural control. In our study, barefoot performance remained completely unaffected even though the practice with rigid soles directly preceded barefoot walking. There was not even a short-lived aftereffect in the trial immediately following the intervention. This observation is in contrast to numerous findings in the motor neuroscience literature on the control and adaptation of target-oriented reaching^21–23^. When reaching movements are exposed to external force fields or visuo-motor rotations, subjects alter their behavior to compensate for these modified environments and, upon removal of the perturbation, they consistently show aftereffects. These aftereffects are interpreted to result from a change in one’s neural controller, specifically an adaptation of one’s internal model of the environment. The striking absence of aftereffects in our study suggests that subjects did not change their neural control strategy. Even if it cannot be completely ruled out that subjects might have adopted a new control strategy in the CF condition, that strategy was entirely context-dependent and did not transfer^28^. It is possible that longer practice with rigid soles^18^ is required to ultimately generalize to other environmental conditions. While conceivable, this remains an open question. At present, the results here indicate that constraining the feet may not be an effective intervention for training or retraining balance. The findings also underscore the notion that paradigms shown to enhance motor learning with the upper extremities do not necessarily translate to tasks involving whole-body coordination. For instance, Domingo and Ferris similarly attempted to enhance learning of walking on a balance beam walking by providing physical assistance^29^ and augmenting error^30^, both interventions that have shown successful effects in upper-limb learning^31–33^. Counter to their predictions, practice with these interventions led to worse performance compared to practicing without any assistance in a beam walking task.

How did the rigid soles alter balance performance? When balancing on a narrow base of support, barefoot or with soles, the ankle’s ability to exert torque on the beam through the foot is limited^9^. Thus, it is not surprising that numerous studies have reported that humans use a hip-dominant strategy to maintain balance when standing on a beam^3,4,8,9,26,34^. Consistent with these studies of standing balance, we similarly observed that subjects used a hip strategy to maintain ML balance when walking on a beam, as indicated by high anticorrelation between the AM of the upper- and lower-body segments^26^. Importantly, this was observed when the arms were allowed to move freely as in real-world conditions. It is important to point out that even though subjects used a hip-dominant strategy during beam walking, this did not mean that the influence of the foot and ankle was negligible as is often presumed when balancing on a narrow beam. In fact, our finding that constraining the feet significantly altered balance behavior manifested otherwise.

Not only did constraining the feet decrease the overall AM magnitude of most individual segments, it also resulted in less anticorrelation between the AM of the upper- and lower-body segments. Though this change in anticorrelation was small, it was significant and was observed in all subjects. As demonstrated in a prior simulation study of a double-inverted pendulum model^26^, the degree of anticorrelation decreases when the overall magnitude of ankle torque increases relative to the magnitude of hip torque. It is important to point out that an increase in the relative contribution of ankle torque with rigid soles could have resulted from an increase in ankle torque, a decrease in hip torque, or a combination of the two. While we cannot discern in our experimental data how the change in relative ankle contribution occurred, we do know that it resulted from altering the physical interaction between the foot and beam. The fact that subjects did not appear to learn a new control strategy during practice with rigid soles suggests that the improvement in balance was the result of a mechanical effect. This was also demonstrated by recent simulation work that illustrated how balance performance can change by altering the dynamics at foot-beam interaction without changing the underlying control policy^35^.

**Figure 12:**
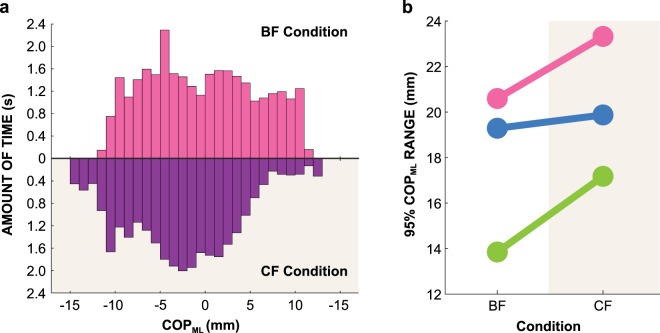
(**a**) Temporal histogram of COP_ML_ values for one example subject while standing on a beam with bare feet (BF) and constrained feet (CF). (**b**) 95% range of center of pressure in the mediolateral direction (COP_ML_) of 3 individual subjects in each condition.

It is important to further scrutinize this mechanical effect of the flat rigid soles as this may point to new approaches for (re-)training balance ability. As the width of the support surface was identical in the barefoot and rigid sole conditions, the flat rigid soles did not increase the maximum torque that could be applied at the ankle. To further examine to what degree subjects utilized the width of the beam, a follow-up experiment measured the center of pressure (COP) as subjects stood on the narrow beam, both with bare feet and constrained feet. Figure 12a shows a temporal histogram of the COP in the mediolateral direction (COP_ML_) for one representative subject in both CF and BF conditions (summed across all trials). The 95% range of the COP in the mediolateral direction (COP_ML_) was increased in the rigid sole (CF) condition compared to the barefoot (BF) condition. Figure 12b presents this increase for three individuals. The rigid soles increased the “effective range” range of COP (i.e., increased the amount of torque that could be applied at the ankle). This could have resulted for several reasons. For instance, while the purpose of the rigid soles was only to constrain foot motion, the fact that the soles were wider than the beam allowed subjects to distribute force over a larger surface area. Thus, the peak pressure did not need to be as high to move the COP to edges of the beam. In contrast, with bare feet, moving the COP to the edges of the beam requires a high concentration of force over a small area at the edge of the beam. It is possible that subjects avoided this strategy due to pain associated with such high pressure. A second possibility is that the rigid soles may have increased the range of the COP that did not result in foot rotation about the beam^20^. While the multiple joint degrees of freedom in each foot may increase control and/or sensing abilities, they also make the foot compliant. Without the soles, moving the COP to edges of the beam could cause the compliant foot to rotate. Therefore, subjects possibly reduced their COP range to avoid this rotation. Future studies would allow distinguishing amongst these possible explanations. Also note that the COP ranges during standing may differ from those during walking. However, these comparisons are beyond the scope of this study.

It is possible that adding the rigid sole changed the friction at the beam surface. However, it is unlikely that this would explain the difference in balance performance between the BF and CF conditions. We observed that subjects would occasionally lose contact with the beam by slipping in trials when wearing the rigid soles, but not in the trials performed with bare feet. This might suggest that the static coefficient of friction of the rigid sole-beam interface was lower than that of the human foot sole-beam interface. Additional mesurements showed that the static coefficient of friction between the rigid soles and wood was approximately 0.34. This meant that an individual with a mass of 68 kg (average of the three subjects) would only induce motion of the sole in the mediolateral direction if the x-component of the ground reaction force (F_x_) was greater than 266.6 N. We measured F_x_ during the barefoot trials in the three subjects. In the BF condition, the maximum magnitudes of F_x_ were 37.6 N, 43.1 N, and 25.4 N for each respective subject, and the 95^th^ percentile values were 18.0 N, 17.8 N, and 10.0 N. Since even the greatest magnitude of F_x_ was substantially lower than the force needed to overcome static friction, the improvement in balance performance with the rigid soles was likely not a consequence of potential differences in the static friction between the rigid soles and the beam and the feet and beam.

Our results showed that manipulating the physical interaction between the feet and beam improved balance performance, suggesting that learning to properly control the interaction between the feet and beam is one of way of improving balance. For instance, it is possible that the subjects who were most skilled at maintaining balance were able to modulate the mechanical impedance of their feet to produce more desirable interactive behavior with the beam. This could explain why Sawers and Ting^18^ observed more muscle synergies in dancers with balance experience. For instance, finer control of the muscles of the lower limbs and feet may enhance the ability to modulate the mechanical impedance of the feet and lower limbs. This also underscores that a simple structure in overt balance behavior is not necessarily indicative of a “simple” controller in the neuromotor system. We may emphasize again that balance proficiency and observed balance performance is not the same. Good balance performance may reflect fast and appropriate anticipation or response to external or internal perturbations, i.e. good balance ability. In addition, successful overt balance performance may be achieved by reducing internal noise and perturbations that reduces the need to correct for perturbations. On the other hand, high variability in balance metrics may also indicate good balance control as good balancers can be riskier. Clearly, teasing apart these issues extends the scope of the current study.

While our results gave clear evidence that adding flat rigid soles can assist balance, this benefit to balance may also come at cost. For instance, Takahashi *et al.*^36^ found that wearing shoes with stiff soles significantly increased the metabolic cost of walking. Ultimately, future work is needed to further understand (1) the influence of the foot and ankle mechanical properties on balance, (2) how expert balancers modulate or compensate for such mechanical properties, and (3) how to leverage the improved performance with rigid soles to improve balance under normal walking conditions. We expect that addressing these open questions will yield promising new insights for enhancing the assistance and rehabilitation of balance.

## Methods

### Subjects

Seven healthy subjects (gender: 2 females and 5 males, age: 28.7 ± 2.5 years, mass: 68.4 ± 10.9 kg, height: 1.74 ± 0.08 m) took part in the experiment. None had any prior experience with the specific experimental task. The experiment conformed to the Declaration of Helsinki. Written informed consent was obtained from all participants according to the protocol approved by the ethical committee at the Medical Department of the Eberhard-Karls-Universität of Tübingen, Germany.

### Experimental protocol

In each trial, the subject walked along a narrow, wooden beam (3.4 cm wide, 3.4 cm high, 4.75 m long; 4 mm wide chamfered edges) at a self-selected speed without stepping off the beam (Fig. 1a). Before the start of each trial, subjects stood with their left foot on the beam and their right foot on the ground. After the experimenter gave the “go”-signal, they also placed their right foot on the beam and began walking. Upon reaching the end of the beam, subjects were instructed to step off, placing their feet on either side of the beam. Subjects did not receive any other instruction on how to walk or how fast they should walk across the beam. They could use all body segments, including arms, as they wished to maintain balance. For data processing, the placement of the right foot on the beam indicated the start of each trial; the last step before stepping off the beam marked the end of the trial. A trial was deemed successful, if the subject remained on the beam for its entire length. If the subject lost balance and had to step on the ground before reaching the end of the beam, the trial was labeled as unsuccessful. After each trial, subjects were allowed to take a short rest if needed.

Each subject was instructed to complete 20 successful trials in each of the following three blocks: Bare Feet–Pre (BF–Pre), Constrained Feet (CF), and Bare Feet–Post (BF–Post). In the BF–Pre and BF–Post blocks, participants walked barefoot (i.e., without shoes and socks); in the CF block, participants performed trials with flat, rigid soles attached to each foot (Fig. 1c). The flat rigid soles were 3D printed using Acrylonitrile Butadiene Styrene (ABS) using a rectangular infill (approximate modulus of elasticity ~ 0.55)^37^ (Fig. 1b). They were designed to be non-deformable under the load of human, lightweight (mass of each sole: 161 g), and slightly larger than all subjects’ feet, which allowed all subjects to wear the same soles (width: 12 cm at widest point, length: 31 cm, depth: 1.2 cm). The flat rigid soles were secured to the bottom of each foot with hook-and-loop straps and reinforced with tape, as illustrated in Fig. 1b. The rigid soles prevented any bending at the midfoot and torsion on the long axis of the foot around the beam (e.g., tarsometatarsal, metatarsophalangeal, and interphalangeal joints). While they did not explicitly eliminate relative motion of the foot joints, these motions were considered negligible. Importantly, these soles did not constrain plantarflexion/dorsiflexion and inversion/eversion of the ankle, allowing ankle torque to act upon the foot.

The static friction coefficient of each sole on the wooden surface was measured to be approximately 0.34. This value was obtained by using the InMotion2 robot (Bionik Labs, Watertown, MA); the robot applied a ramp force to a loaded sole (157 N) parallel to the surface and recorded the maximum force before a displacement of the robot (and consequently the sole) was detected. In principle, it would be desirable for the sole-beam and foot-beam interface to have the same friction coefficient to eliminate differences from this mechanical property. However, the static friction coefficient of the subjects’ foot-beam interface was hard if not impossible to estimate from each individual directly. It is also likely that each subject had a different coefficient due to different surface properties of the skin and different shapes of the foot. However, we observed that subjects would occasionally lose contact with the beam by slipping in trials when wearing the rigid soles, but not in the trials performed with bare feet. This might suggest that the static friction coefficient of the rigid sole-beam interface was lower than that of the human foot-beam interface. However, results from the additional experiment measuring ground reaction forces showed that the forces subjects exerted in the mediolateral direction were small enough such that potential differences in static friction should have had minimal effect on balance performance (see Discussion for further details).

### Motion capture data collection

For each trial, 3D whole-body motion capture data was collected using a 10-camera motion capture system (Vicon, Oxford, UK) at a sampling rate of 100 Hz. Reflective markers were placed on the subjects’ bodies following Vicon’s Plug-In Gait marker set (Fig. 1a). The origin of the lab coordinate frame was set to the start of the beam, with its y-axis aligned along the beam, and its x-axis perpendicular to the beam. Commercial Vicon software was used to reconstruct and label the markers and to interpolate between short missing segments in the 3D marker trajectories.

Based on the subjects’ self-reported height and weight, subject-specific dynamic models were fit to the 3D marker trajectories using C-Motion Visual3D software (Germantown, MD). The Plug-In Gait model consisted of 15 rigid body segments (Table 1). The dependent measures for each trial were calculated using the model-based data exported from Visual3D that were subsequently analyzed using custom scripts in Matlab (The Mathworks, Natick, MA) as described in detail below.

### Dependent measures

#### Number of failed trials

The number of failed trials for each subject in each block presented a course-grained measure for overall task performance.

#### Center of mass velocity (COM-V)

For each sample in a given trial, the velocity of the center of mass in the ML-direction (i.e., $x$-direction) (COM-V) was approximated by computing the backward difference of the COM position values. The COM position in the ML-direction at sample *t*, denoted as ${r}_{x}(t)$, was calculated as follows:

$${r}_{x}(t)=\frac{{\sum }_{i=1}^{15}{m}_{i}{c}_{i,x}(t)}{{\sum }_{i=1}^{15}{m}_{i}}$$

where ${m}_{i}$ denoted the mass of the $i$^th^ segment, and ${c}_{i,x}(t)$ was the position of the $i$^th^ segment’s COM in the $x$-direction at sample $t$. For each trial, the RMS of COM-V was calculated using the COM-V values from samples from only the middle 67% (i.e., two-thirds) of each trial to avoid any possible transients or fatigue effects in the dependent measures. This elimination of transient effects was also applied to all subsequent root mean square (RMS) calculations. For each subject, the average RMS COM-V of all trials in each block was calculated to assess change in balance performance across blocks.

#### Individual body segment angular momentum (AM)

In each trial, the angular momentum (AM) from each of the individual 15 body segments was calculated. In a given trial, the AM of each $i$-th body segment at each sample $t$ about the beam axis (i.e., $y$-axis), denoted by ${L}_{i,y}(t)$, was calculated using the following equation:

$${L}_{i,y}(t)=[\begin{array}{l}{c}_{i,x}(t)\\ 0\\ {c}_{i,z}(t)\end{array}]\times {m}_{i}[\begin{array}{l}{v}_{i,x}(t)\\ 0\\ {v}_{i,z}(t)\end{array}]+{(I\omega )}_{i,y}(t)$$

where ${c}_{i,x}(t)$ and ${c}_{i,z}(t)$ are the positions and ${v}_{i,x}(t)$ and ${v}_{i,z}(t)$ are the linear velocities of the $i$-th segment’s center of mass in the respective $x$- and $z$-directions at sample $t$. ${(I\omega )}_{i,y}(t)$ was the angular momentum of the $i$-th segment about its center of mass expressed in the lab coordinate frame. Note that AM of segment $i$ about its center of mass was first calculated in its local coordinate frame and then transformed into the lab coordinate frame using the Visual3D software. For each trial, the RMS of the AM from each body segment was then calculated, again only for samples from the middle 67% of each trial.

#### Whole-body angular momentum (WB-AM)

WB-AM about the beam (i.e., $y$-axis) at sample $t$ in a given trial, denoted by ${L}_{{\rm{wb}},y}(t),\,$was calculated by summing the AM of all individual body segments as follows:

$${L}_{{\rm{wb}},y}(t)=\mathop{\sum }\limits_{i=1}^{15}{L}_{i,y}(t).$$

For each trial, the RMS of the AM from each body segment was then calculated using only samples from the middle 67% of each trial.

#### Correlation of upper- and lower-body angular momentum (CORR-AM)

The correlation coefficient at lag-0 was calculated between the lower-body and upper-body AM signals in each trial. Lower- and upper-body AM about the beam (i.e., $y$-axis) at sample $t$ in a given trial, denoted respectively by ${L}_{{\rm{lb}},y}(t)$ and ${L}_{{\rm{ub}},{\rm{y}}}(t)$, were calculated by summing the AM of the lower- and upper-body segments, respectively, as follows:

$${L}_{{\rm{lb}},{\rm{y}}}(t)=\mathop{\sum }\limits_{i=1}^{7}{L}_{i,y}(t)$$

$${L}_{{\rm{ub}},{\rm{y}}}(t)=\mathop{\sum }\limits_{i=8}^{15}{L}_{i,y}(t)$$

Only the middle 67% of samples in the signals of each trial were used to calculate the correlation coefficient.

### Statistical analyses

To assess change in balance performance across blocks, the average of the RMS of COM-V, RMS of WB-AM, RMS of AM from individual body segments, and the CORR-AM across trials was calculated for each block and subject. These measures, along with the number of failed trials, were then subjected to a one-way ANOVA with block (BF-Pre, CF, BF-Post) as the within-subjects factor. When the main effect of block was found to be significant, planned comparisons in the form of pairwise t-tests were conducted. The assumption of sphericity in the observed dependent measures was not violated as indicated by the non-significant results from Mauchly’s tests conducted for all ANOVAs.

To determine whether practice in the constrained foot block had an immediate influence on subsequent balance performance with bare feet, pairwise t-tests were conducted to compare RMS of COM-V, RMS of WB-AM, and CORR-AM in the last trial of the BF-Post block and the first trial of the BF-Post block average RMS of COM-V.

In all statistical tests, the significance level was set to p = 0.05. The ANOVAs were performed using SPSS Statistics for Windows, Version 24.0 (IBM Corporation, Armonk, NY), and the pairwise t-tests were performed using MATLAB, Version 2016b (The Mathworks, Natick, MA).

### Methods for additional experiment measuring center of pressure and ground reaction force

Three healthy subjects (gender: 2 females and 1 male, age: 42.0 ± 16.5 years, mass: 64.9 ± 13.4 kg, height: 1.67 ± 0.14 m) took part in the experiment. In each trial, subjects were instructed to stand on the beam with their feet in tandem for 30 s. Each subject performed a total of six trials, three with bare feet (BF condition) and three with feet constrained (CF condition). The flat rigid soles were the same as those used in the main experiment. The beam had the same dimensions as the one used for walking. It was placed on a 600 × 800 mm force plate (AMTI, Watertown, MA), which was used to measure the center of pressure (COP) displacement and ground reaction force at a rate of 500 Hz.

The position of the COP in the mediolateral direction (i.e., $x$-direction) (COP_ML_) and anterior-posterior direction (i.e., $y$-direction) (COP_AP_) on the beam surface at sample $t$ in a given trial, denoted by ${p}_{{\rm{b}},x}(t)$ and ${p}_{{\rm{b}},y}(t)$ respectively, were calculated using the following equations:

$${p}_{{\rm{b}},x}(t)={p}_{{\rm{f}},x}(t)+h\frac{{F}_{{\rm{f}},x}(t)}{{F}_{{\rm{f}},z}(t)}$$

$${p}_{{\rm{b}},y}(t)={p}_{{\rm{f}},y}(t)+h\frac{{F}_{{\rm{f}},y}(t)}{{F}_{{\rm{f}},z}(t)}$$

where ${p}_{f,x}(t)$ and ${p}_{f,x}(t)$ were the $x$- and $y$-positions of the COP measured at the surface of the force plate, ${F}_{{\rm{f}},x}(t)$, ${F}_{{\rm{f}},y}(t)$, and ${F}_{{\rm{f}},z}(t)$ were the $x$, $y$, and $z$-components of the ground reaction force at sample $t$, and $h$ was the vertical height of the beam. In some trials, subjects lost contact with the beam before 30 s, but all subjects were able to maintain balance for at least 10 s in all trials. Thus, only the first 10 s of each trial were analyzed. For each trial, the 95% range (i.e., range between the 2.5^th^ and 97.5^th^ percentile) and the median of the COP_ML_ from the samples in the first 10 s of all trials in each condition was calculated.

## Data Availability

The data will be made available on the website of Dagmar Sternad: https://web.northeastern.edu/actionlab/research/.
